# Long distance seawater intrusion through a karst conduit network in the Woodville Karst Plain, Florida

**DOI:** 10.1038/srep32235

**Published:** 2016-08-25

**Authors:** Zexuan Xu, Seth Willis Bassett, Bill Hu, Scott Barrett Dyer

**Affiliations:** 1 Department of Earth, Ocean and Atmosphere Science, Florida State University, Tallahassee, Florida 32306, USA; 2 Florida Geological Survey, Tallahassee, Florida 32303, USA; 3 Department of Ecology, Jinan University, Guangzhou, Guangdong, China; 4 Climate and Ecosystem Sciences Division, Lawrence Berkeley National Laboratory, Berkeley, California 94720, USA

## Abstract

Five periods of increased electrical conductivity have been found in the karst conduits supplying one of the largest first magnitude springs in Florida with water. Numerous well-developed conduit networks are distributed in the Woodville Karst Plain (WKP), Florida and connected to the Gulf of Mexico. A composite analysis of precipitation and electrical conductivity data provides strong evidence that the increases in conductivity are directly tied to seawater intrusion moving inland and traveling 11 miles against the prevailing regional hydraulic gradient from from Spring Creek Spring Complex (SCSC), a group of submarine springs at the Gulf Coast. A geochemical analysis of samples from the spring vent rules out anthropogenic contamination and upwelling regional recharge from the deep aquifer as sources of the rising conductivity. The interpretation is supported by the conceptual model established by prior researchers working to characterize the study area. This paper documents the first and longest case of seawater intrusion in the WKP, and also indicates significant possibility of seawater contamination through subsurface conduit networks in a coastal karst aquifer.

Seawater intrusion is an important and emerging topic in environmental research, particularly in light of its tangible and global environmental impacts. Soil salinization, marine and estuarine ecological change, and groundwater contamination are all significant challenges in coastal regions^1^. Water resources and ecological systems are extremely sensitive to seawater intrusion, and mixing less than 1% of seawater (250 mg/L chloride) by volume with drinking supplies can render freshwater aquifers non-potable^2^. Karst aquifers are among the most vulnerable types of coastal systems susceptible to the impacts of seawater intrusion, and they are enormously important resources: karst aquifers underlie 10–20% of the Earth’s landmass and supply potable water to nearly 25% of the world’s population^2^,^3^. In the US alone, karst aquifers supply almost 52% of all bedrock freshwater aquifer withdrawals annually^4^,^5^.

In a porous medium aquifer, the extent of freshwater/seawater mixing interface can be described by the Ghyben-Herzberg equation and Hubbert relationship^6^,^7^. Custodio^8^ studied several controlling factors for seawater intrusion into freshwater coastal systems, including geologic and lithologic heterogeneity, localized surface recharge, paleo-hydrogeological conditions and anthropogenic influences. Werner *et al*.^9^ concluded that climate variations, groundwater pumping and fluctuating sea levels were also important factors in determining the distribution of seawater intrusion in a coastal aquifer. Depression cones of groundwater level due to over-pumping near coastal metropolitan areas can exacerbate seawater intrusion by reducing the hydraulic pressure of the freshwater aquifer and thereby lessening its ability to retard intruding seawater^10^,^11^.

Unfortunately, classic hydrogeologic knowledge is not appropriate, or at least incomplete, in many karstic aquifers, and while seawater intrusion is only observed near the shoreline in most coastal aquifers, this is not the case in karst^9^. Karst aquifer systems are characterized by dissolution, high permeability and high effective porosity, all of which serve to make them among the most productive types of aquifer systems in the world^12^. The open and complex nature of karst systems makes characterizing the behavior of coastal karst aquifers one of the most complex and challenging tasks in hydrogeology. Despite the global importance of karst aquifers to human activity, the complicated mechanics of seawater intrusion in a karst setting remain poorly understood.

In the coastal setting, many karst aquifers have an extensive and well-developed subterranean network of conduits that terminate in the sea^13^. Groundwater flow and solute transport in the conduit networks are significantly faster than those in the surrounding matrix domain, producing a complex, dual-permeability flow through the matrix and conduit components of the aquifer^14^,^15^,^16^. Conduit systems serve to facilitate the rapid transport of seawater many miles inland, and long-distance seawater intrusion has been observed in the karst coastal aquifers in the Yucatan Peninsula, where a well-developed conduit network connects inland sinkholes, fractures, and springs directly to the Gulf of Mexico^13^,^17^,^18^.

A well-developed conduit system also serves to complicate the fate of seawater once it enters the coastal aquifer. The complex interchange between the conduit and matrix components of a karst aquifer determines the ultimate impacts and extents of seawater intrusion^19^,^20^. During high-flow events, contaminants in the conduit system can be transported rapidly and pushed into the carbonate matrix when the relative water pressure in the conduit is higher than the pressure in the matrix component. During low-flow events, this process is reversed, with contaminants migrating from the surrounding matrix and back into the conduits^21^,^22^.

More than fifty years of research have been devoted to coastal groundwater flow and transport processes, but the processes involved in seawater intrusion in karst aquifers remains a challenging topic, particularly with regard to field measurements and modeling evaluation^9^. This paper reports the most extended case of seawater intrusion documented in the WKP using a comprehensive interpretation of conceptual site models, electrical conductivity, rainfall, gage, flow, and geochemical data. The results of this study both illustrates the complexities inherent to studying karst aquifers, and demonstrates that under the right local conditions seawater can be transported 11 miles inland against the prevailing regional hydrologic gradient via a well-developed conduit system.

## Study Site

The study site is located in the southwestern portion of the Woodville Karst Plain (WKP), Florida, USA. The WKP is bounded to the north by the Cody Scarp, to the west by the Apalachicola National Forest, and to the south by the Gulf of Mexico. The WKP is a highly karstified, gently rolling surface of unconsolidated and porous Pleistocene sands overlying Eocene, Oligocene and Miocene limestone^23^. Permeable and heterogeneous carbonate units of porous limestone are observed throughout the study area, including the highly permeable subsurface fractures and an extensive and well-developed conduit system^12^,^19^.

The predominant first magnitude spring in the study area is Wakulla Spring, one of the largest and most dramatic of Florida’s freshwater springs^24^. Wakulla Spring is located 11 miles north of the Gulf Coast with an annual discharge of 400 *ft*^3^/s into the 14.3 mile long Wakulla River^19^,^24^. The main vent is approximately five feet above sea level and forms a horizontal ellipse measuring 50 ft by 82 ft that is connected to an extensive horizontal conduit system with an average depth of about 200 ft below land surface (BLS) proximate to the main vent. The depths of the conduit system increase as one moves horizontally away from the vent, eventually reaching over 300 ft BLS^24^,^25^.

**Figure 1:**
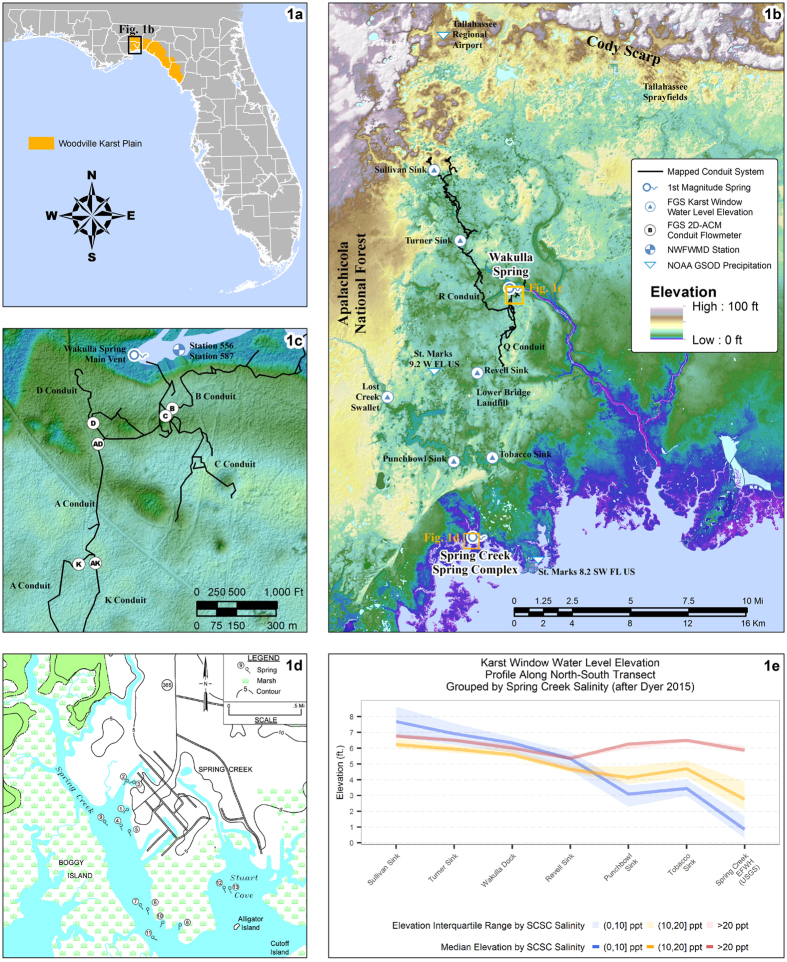
(**a**) Locations of the Woodville Karst Plain and the study site; (**b**) Map of the Woodville Karst Plain, showing the locations of features of note within the study area; (**c**) Locations of Falmouth 2D-ACM meters and Wakulla Spring cave system; (**d**) Locations of Spring Creek Spring vents, from Lane 21; (**e**) Groundwater elevation profile in major karst windows grouped by Spring Creek salinity, from 22. Figure 1(a–c) were created using ArcMap version 10.3.1, copyright and licensed by ESRI, http://desktop.arcgis.com/en/; (**d**) is a reprint from Lane 21; (**e**) was created using R version 3.2.3, licensed under GUN Public License 2&3 (GPL-2&3), https://www.R-project.org/. All maps and data in figures were created using data acquired by the State of Florida those are also in the public domain and not subject to copyright.

The Wakulla conduit system is one of the most extensively mapped underwater cave systems in the world. The Woodville Karst Plain Project (WKPP) divers have successfully mapped more than 37 miles of human-navigable conduits, with each major conduit identified by an alphabetic letter designation^26^ (Fig. 1c). Starting at the main vent, the conduit system runs southwest until it meets the confluence of the north-oriented B conduit and the south-oriented C conduit (Fig. 1). The main conduit then extends westward until it bifurcates at the junction of south-north oriented A conduit and the north-south oriented D conduit. The A conduit continues to the junction of the A and K conduits. The south-oriented K conduit eventually intersects the R conduit which runs north into Leon County and is one of the major sources of surficial recharge at Wakulla Spring. South of the A conduit is the Q conduit, which trends southward towards the Gulf Coast and is presumed to connect with the Spring Creek conduit system.

The other first magnitude spring of note in the study area is the Spring Creek Springs Complex (SCSC), a group of 14 submarine springs located in the Gulf of Mexico^27^ (Fig. 1d). The SCSC vents are supplied by an extensive, interconnected and highly complex conduit network. The depths of the vents at SCSC were estimated by Davis and Verdi^28^ to be approximately 300 ft deep, based on the assumption that they descend to a similar depth to the conduits at Wakulla Spring. Tracer tests conducted by the Florida Geological Survey (FGS) indicate that the SCSC conduit system bifurcates north of the vents, with one branch trending north to Wakulla Spring, and another trending northwest to Lost Creek Swallet^26^,^29^,^30^.

It has become apparent that SCSC, despite having a large aggregate outflow during periods of high rainfall, can become a significant site of seawater intrusion during low rainfall periods. The United States Geological Survey (USGS) and the FGS have verified that under conditions of extended low rainfall or during periods of abnormally high tidal surges, SCSC can reverse flow and begin siphoning brackish seawater from the SCSC embayment into the inland aquifer. Lane *et al*.^27^ notes that the potentiometric surface at SCSC is in “tenuous balance with the marine environment”. Davis^28^ claimed the rising sea level adversely impacted the balance between the freshwater aquifer system and the marine waters of the Gulf. Xu *et al*.^31^ quantitatively calculated the impact of sea level rise on spring discharges and groundwater flow pattern in the WKP.

## Conceptual Model

This study builds upon the conceptual models established by Dyer^29^, Davis and Verdi^28^, Schmidt *et al*.^32^, the FGS^33^, Loper *et al*.^34^, and Xu *et al*.^31^ among many others, in order to explain how seawater can be transported against the regional hydraulic gradient 11 miles inland to Wakulla Spring. Taken in aggregate, these earlier works show that the discharge of Wakulla Spring and SCSC are reliant upon conditions at the margins of the WKP. Groundwater recharge flows to Wakulla Spring from the north and west at the regional scale, and the topographic boundaries of the Cody Scarp and Apalachicola National Forest coincide with a relatively steep, upward sloping potentiometric gradient. Tides also play a role, with both the matrix and conduit components of the aquifer proximal to the Gulf of Mexico at SCSC; tidal cycles are clearly evident in both karst window water levels and monitor wells connected to the conduit system throughout the springshed. Thus, the area immediately southwest of Wakulla Spring is bounded on the west and north by regional groundwater pressure, to the east by the Wakulla River, and to the south by the Gulf of Mexico.

Within this wider hydrogeologic setting, Wakulla Spring and SCSC exhibit a strong coupled flow behavior, and flow conditions at one spring are closely related to the other. The constant hydraulic head at Wakulla Spring is only 5.0 ft above mean sea level, and acts as a constant head boundary for the local potentiometric surface. Comparatively, the estimated equivalent freshwater head at SCSC is highly variable and changes dynamically both with the tides and with the elevation of the local potentiometric surface: during periods when SCSC is discharging it can be as low as 0~1 ft, versus 7.5 ft when the SCSC conduit system is filled with seawater^28^,^31^. Multiple dye trace studies conducted by the FGS show that water entering the conduit system at Lost Creek Swallet transits southward when SCSC is flowing, but transits northward to Wakulla Spring following low rainfall periods when SCSC ceases to flow and freshwater normally flowing to SCSC can no longer find an outlet at the coast^24^,^30^. This inland freshwater is subsequently rerouted via the conduit system to the vent at Wakulla Spring. The rerouting of water results in the increased discharge at Wakulla Spring, as noted by Davis and Verdi^28^. Numerical modeling conducted by Xu *et al*.^31^ has confirmed the speculation of Davis and Verdi^28^ that rising sea level can cause the water flowing to SCSC to shift inland to Wakulla Spring.

Dyer^29^ found that water level elevations increase as much as 3 ft in the karst windows south of Revell Sink during periods of negative discharge at Spring Creek (Fig. 1e). This work shows that when SCSC is no longer discharging, the water level elevations in the karst windows south of Wakulla Spring are at higher elevations than the vent at Wakulla Spring than they are when SCSC is discharging fresh water. Thus, under low rainfall conditions, the potentiometric gradient in conduits southwest of Wakulla Spring reverses and runs against the prevailing regional gradient, and the constant low hydraulic head at Wakulla Spring attracts water that was formerly flowing south to SCSC.

## Method

In late 2003, FGS installed six Falmouth 2D-ACM acoustic flowmeters in the conduits proximal to the main vent at Wakulla Spring. These flowmeters are located between 280–300 ft below land surface in the B, C, D, AD, AK, and K conduits (Fig. 1c). In late 2009, three additional meters were installed at three sites south of the Wakulla vent: one meter was placed in the conduit beneath Revell Sink approximately four miles southwest of Wakulla Spring, and two meters were installed at SCSC Vent #1 and SCSC Vent #10. These nine flowmeters record five parameters at 15 minute or hourly intervals: average speed of flow (ASPD), electrical conductivity (COND), water pressure (PRES), water temperature (TEMP), and average flow direction (AVDIR). These meters are used in conjunction with a network of *In Situ* Level Troll devices measuring the water levels in karst windows connected to the deeper conduit system.

Several additional datasets were utilized to augment the conduit flowmeter data. The USGS installed a gage at Spring Creek in 2007 recording gage height, electrical conductivity, salinity and the aggregate discharge for 12 of the 14 vents at SCSC. Precipitation data from the National Oceanic and Atmospheric Administration (NOAA) Global Historical Climatology Network (GHCN) dataset^35^ were used to track rainfall at the nearby Tallahassee Regional Airport. Two Northwest Florida Water Management District (NWFWMD) stations, #587 and #556, were used to measure the aggregate electrical conductivity and discharge at the Wakulla vent and verify the accuracy of the 2D-ACM conductivity data. Finally, NWFWMD provided a dataset containing intermittent, laboratory geochemical analyses conducted on grab samples taken by the NWFWMD, FGS, USGS, and other government agencies at the vent of Wakulla Spring.

## Results

**Figure 2:**
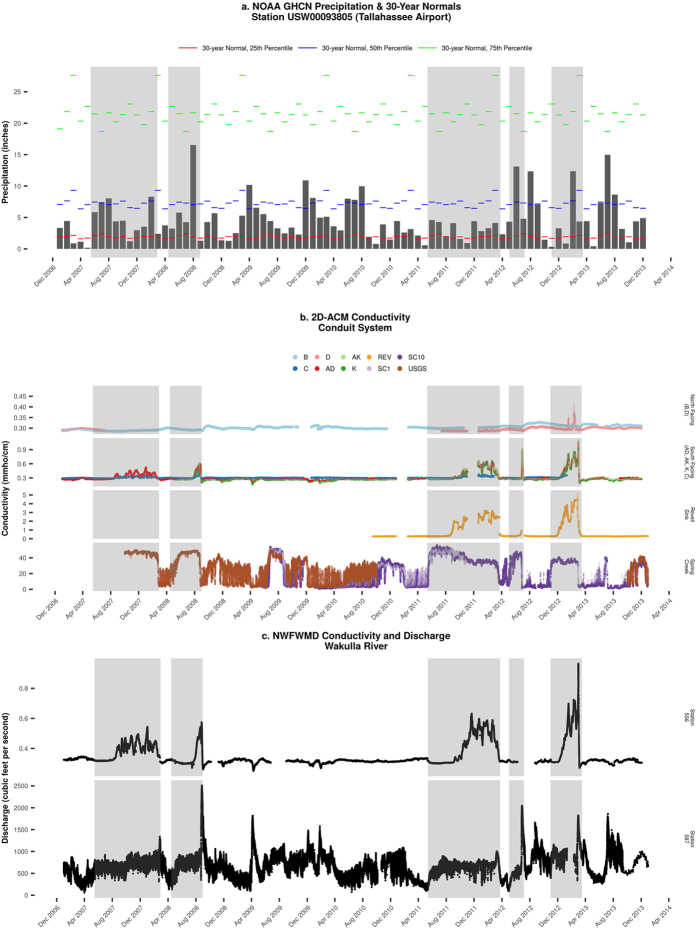
(**a**) NOAA weekly precipitation totals; (**b**) Falmouth 2D-ACM electrical conductivity data in the conduit system; (**c**) Electrical conductivity and discharge at Wakulla River.

**Figure 3:**
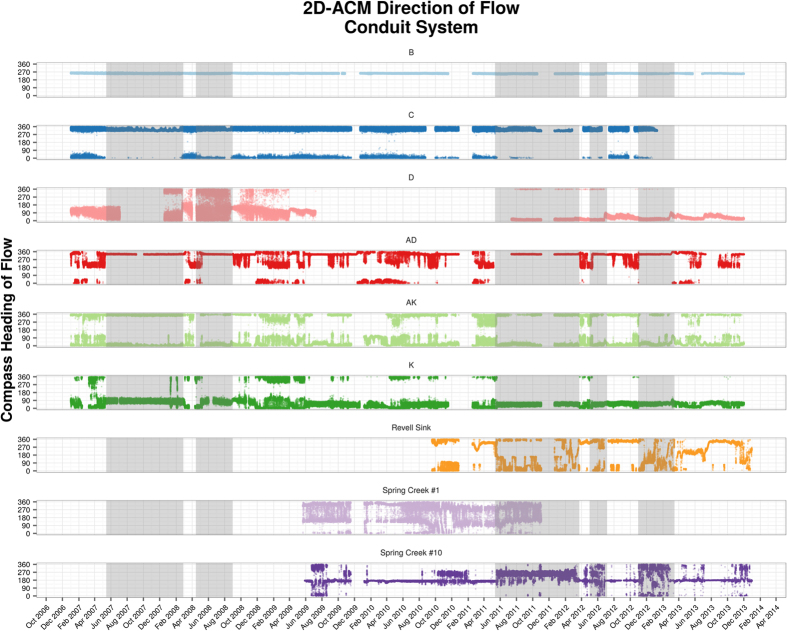
2D-ACM direction of flow data for the Wakulla and Spring Creek conduit systems. Note that the y-axis, representing the compass heading of flow, is topologically discontinuous, and that the bottom of the graph (0 degrees) and top of the graph (360 degrees) both represent northward-flow.

Figure 2a shows the monthly precipitation at the NOAA GHCN station located at the Tallahassee Airport, along with the NOAA 30-year normal precipitation percentiles for this station. Figure 2b illustrates the time-series electrical conductivity data collected by 2D-ACM flowmeters in the B, D, AD, AK, K, and C conduits, the conduit beneath Revell Sink, and both the FGS and the USGS data recorded at SCSC. Figure 2c displays the NWFWMD discharge and conductivity measured at the main vent at Wakulla Spring. Figure 3 shows the average flow direction in the subsurface conduit system by the 2D-ACM flowmeters.

Importantly, the 2D-ACM data illustrates that the nominal electrical conductivity in the conduits remains relatively constant 0.28–0.30 mmho/cm during most of the observation period (Fig. 2b). There are, however, five distinctive episodes of rising conductivity values exceeding the nominal background conductivity values in the conduit system near Wakulla Spring (Table 1). These five periods of rising conductivity are immediately attributable to periods of low rainfall, as each period of rising conductivity occurred after a month or more of rainfall near or below the 25th percentile of the NOAA 30-year normal values (Fig. 2a).

The beginning and end of each period was determined based on the conductivity at SCSC for all periods except Period 1. The beginning date of each period (except Period 1) was determined when SCSC conductivity values began to trend upwards from a baseline conductivity of less than 10 mmho/cm, indicating the water in the SCSC vents were beginning the transition from freshwater to seawater. Similarly, the end date for each period (except Period 1) was determined when SCSC conductivity values returned to a stable value of less than 10 mmho/cm after transitioning from conductivity values above 30 mmho/cm. For Period 1, no data was available for the SCSC vents, so the start and end dates were picked based upon the aggregate cubic discharge at the Wakulla Vent, as measured by NWFWMD #587. The beginning and end date for this period were determined as the point where the aggregate discharge at Wakulla began to trend upward to a level greater than 500  cubic feet per second. The logic for this choice was based on the conceptual model outlined in the preceding sections, in particular the coupled flow behavior described by Davis and Verdi^28^, where a lack of flow at SCSC produces a stable and reliable increase in the flow at Wakulla.

**Figure 4:**
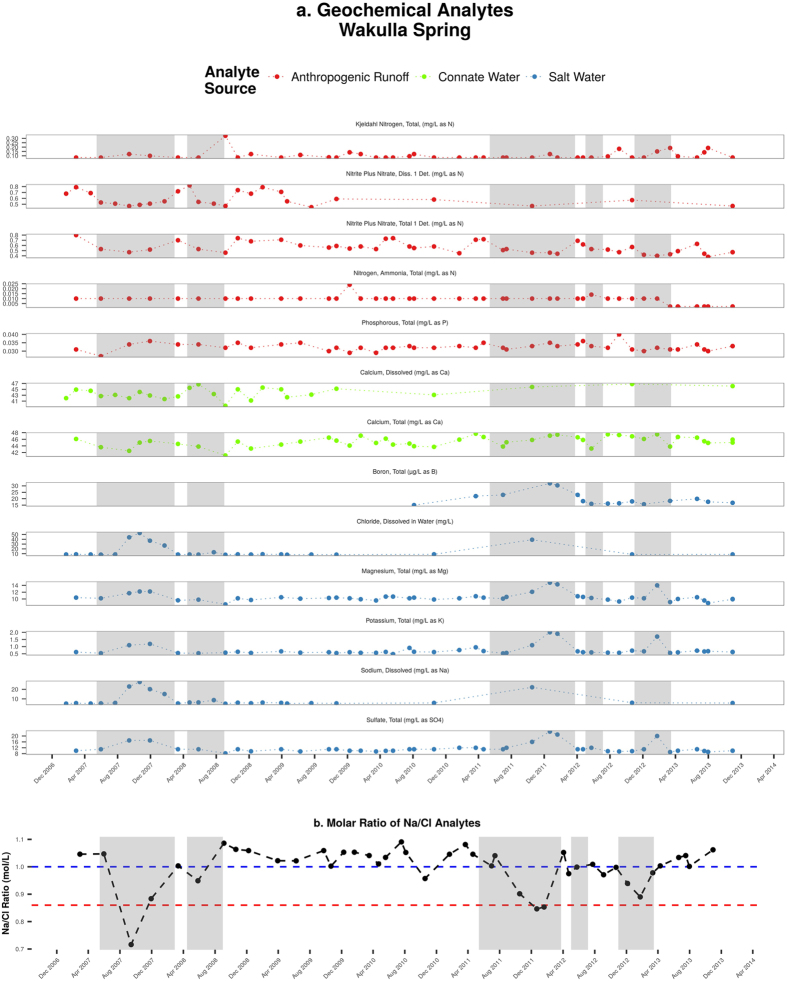
(**a**) Geochemical analytes at Wakulla Spring, including sources of anthropogenic runoff, upwelling recharge connate water and seawater; (**b**) Molar ratio of Na/Cl analytes indicating seawater intrusion.

For the sake of convenient comparisons and graphical clarity, four of the five periods have been highlighted in gray in all parts of Figs 2 and 4. The following discussion section focuses on these five periods, labeled as Period 1–5, respectively.

## Discussion

Three lines of evidence derived from the dataset of 2D-ACM flowmeters indicate that the rising conductivity observations in the conduit system are indicative of coastal seawater intrusion: the spatial orientation of the conduits in which the rising conductivity values are measured, the geospatial pattern of maximum conductivity within the conduits, and the spatiotemporal pattern associated with the periods of rising conductivity values, which are discussed in the following paragraphs. The reversal flow during the highlighted periods are clearly shown by the flow direction data (Fig. 3). Cross comparison with the geochemical dataset supports our interpretation of the conductivity and direction of flow data by ruling out anthropogenic contamination or upwelling groundwater as source of increased conductivity values.

The spatial orientation of the individual conduits provides strong evidence that the increases in conductivity have their origins in coastal seawater intrusion. The conduits conducting water from the north (B & D) show little to no increases in conductivity during most of the identified periods, whereas the conduits transporting water from the south (AD, AK, C & K) all exhibit a similar pattern of rising conductivity (Fig. 2b). The increased conductivity values in the northward-facing D conduit during Periods 4 and 5 are posited to be related to the sensor’s location relative to the conduit geometry. D tunnel is to the north of conduit system connecting to the south, and the slightly increased conductivity values in D conduit could be the result of more conductive water flowing northward past the A-D junction and continuing to the D conduit sensor (Figs 1c and 2b).

The geospatial pattern of the maximum conductivity values within the conduit system also indicates that seawater sourced from the Gulf of Mexico is slowly migrating northward in a manner that agrees with the conceptual model outlined above. Examining the maximum conductivity values for each station, there is a distinct pattern of decreasing conductivity values as one moves landward (north) from SCSC: 50 mmho/cm at SCSC; 5 mmho/cm at Revell Sink; 1 mmho/cm at AK and K conduits; and 0.5 mmho/cm in C conduit, which can be found on the y-axis scale of Fig. 2b.

The spatiotemporal pattern of rising and falling conductivity also clearly tracks from south to north over time and space. The rising conductivity at SCSC indicates that the system has ceased to discharge freshwater and begun to siphon seawater. As the conductivity at SCSC increases to highly saline (>30 mmho/cm), the conductivity beneath Revell Sink begins to increase approximately 28–77 days after the initial flow reversal at SCSC (Fig. 2b). The increases in conductivity at the AK/K/AD trio lag behind Revell Sink by 7–11 days and behind SCSC by 35–110 days. The most northern of the conduits transporting water from the south (C) lags behind all of the other stations, with increases in conductivity occurring 5–24 days after the AK/K/AD trio and 39–115 days after SCSC (Table 2).

There is also a discernible spatiotemporal pattern to the return to nominal conductivity in the conduit system. Falling conductivity values are generally faster than rising process, following with heavy rainfall events that provide sufficient recharge to reestablish flow at SCSC. Particularly important here is the allogenic recharge from the Apalachicola National Forest, which is funneled into the Lost Creek Swallet in enough volume to force the denser seawater out of the SCSC conduit systems^28^. Thus, we see a reversal of the spatiotemporal trend outlined in the preceding paragraph, beginning with a resumption of freshwater flow at SCSC. This resumption of flow is followed in turn by a return to nominal conductivity that tracks from north to south: first C conduit, then the AD/AK/K trio, and finally Revell Sink return to their normal values in turn. The return to nominal conductivity occurs significantly faster than the initial increase, with a lag time of only 1–11 days between AK/AD/C/K and Revell Sink (Table 3).

The only exception to the spatiotemporal patterns outlined above is Period 4, which shows an intense spike in conductivity in the conduits near the vents that lasts a very short duration. This period reflects the confluence of two abnormal events: the historic drought of 2011 and the extreme rainfall and storm surge produced by Tropical Storm Debby in late June, 2012. We theorize that the large storm surge generated by Debby, combined with the long period of drought prior to the storm, forced the conductive water proximate to the coast to migrate rapidly northward. This was followed by a quick return to nominal conductivity as Debby moved inland and dropped progressively larger amounts of rainfall over the region.

The 2D-ACM meters also document the average flow direction (AVDIR) in the conduit systems and provided additional information of the regional flow pattern during the periods of interest (Fig. 3). During these periods, the directional data clearly indicate that flow has reversed at the coastal SCSC conduit, as values trend away from due south. Importantly, the AVDIR observations at AD, AK and K conduits exhibit significantly different pattern during the highlighted periods, and indicate a reversal in flow pattern south of the Wakulla Spring. During periods of normal rainfall, these conduits will generally alternate between flowing north and flowing south, but during the highlighted periods, these conduits cease to flow south almost entirely altogether. The AVDIR measurements in B, C and D conduits remain constant all year around, as these conduits are primarily carrying water sourced from groundwater recharge. Flow direction patterns at Revell are poorly understood at the present time, as the conduit structure and geometry in close proximity to this sensor are insufficiently explored; additionally, this meter sits in different tier of the conduit system, 100 ft closer to land surface than the conduits near the Wakulla vent.

However, the 2D-ACM conductivity and flow direction measurements do not provide enough evidence that the conductive water being measured is saline water sourced from the Gulf of Mexico. Two alternative explanations are also possible: the conductivity spikes could be related to either anthropogenic contamination or upwelling regional groundwater. In order to consider these possibilities, the geochemical dataset provided by NWFWMD is examined (Fig. 4a).

Deep regional groundwater recharge (i.e., upwelling connate water) generally has a relatively higher electrical conductivity when compared to groundwater closer to the surface. This high conductivity is due to the deep groundwater’s longer residence time and long-term geochemical interaction with the limestone matrix^36^. Two parameters recorded in the NWFWMD dataset, total calcium and total dissolved calcium are associated with upwelling groundwater^37^. During the period of study, the concentration of calcium analytes remained relatively constant, indicating that they offered a negligible contribution to increases in total conductivity. We consider it a low likelihood that the increases in conductivity observed in the conduit system are related to upwelling groundwater.

The other potential source of conductive water in the study site is considered as water sourced from anthropogenic runoff. There are at least two major sources of potential anthropogenic contamination in the study area: the Southeast Farm Wastewater Reuse Facility, a series of effluent sprayfields more colloquially known as the Tallahassee Sprayfield; and the Lower Bridge Landfill Facility (LBLF), a now closed general landfill facility to the south of Wakulla Spring (Fig. 1b). Tracer studies have shown that dye introduced into monitor wells at the sprayfield will transit to the main Wakulla vent^26^,^30^, and the LBLF sits close to both the Q conduit and the Revell Sink monitoring site. Additionally, the geometry of the conduit system allows surface runoff from Tallahassee to enter the main conduit system from south of the vent, as the R conduit connects Wakulla Spring with the southern portion of Leon County but joins the main conduit system south of the AK/K junction (Fig. 1b). Nutrient and organic components associated with both of these anthropogenic sources of contamination include nitrates, organic carbon, Kjeldahl nitrogen, ammonia and phosphorous. Examination of Fig. 4a reveals that these anthropogenic analytes show no periodic increases which correlate with the periods of high conductivity. The organic and nutrient components also show a characteristic increase during the summer months when the Sprayfield is in heavy use, which is consistent with the effects of the Sprayfield on the water quality at Wakulla Spring. Conversely, the increases in conductivity seen in the conduits occurred predominantly during the winter months, when SCSC has typically ceased to flow. Therefore, we conclude that there is low likelihood that anthropogenic sources are responsible for the observed rising conductivity values.

There is also a striking similarity between the rising conductivity values in the conduit system and analytes associated with seawater. The analytes associated with seawater, including chlorides, magnesium, potassium, sodium, boron and sulfates^38^, are elevated during the same periods when SCSC has ceased to flow and conductivity is on the rise in the conduit system. The concentrations of the seawater analytes then return to lower values during periods when SCSC is flowing and conductivity in the conduits returns to nominal. The molar ratio of sodium to chloride can also be used to determine the source of water^39^,^40^. Vengosh and Rosenthal^40^ state that the molar ration of Na to Cl when seawater (Na/Cl = 0.86) and freshwater (Na/Cl > 1) mix lies between 0.86 to 1. However, carbonate karst aquifer usually result in lower Na/Cl ratios relative to marine value due to sodium retention by the exchange for calcium and magnesium^39^. The Na/Cl ratios exhibit relatively low values during low rainfall periods, and are correlated with the rising conductivity level (Fig. 4b). This provides additional evidence that the increases in conductivity seen in the vent are associated with seawater intrusion sourced from SCSC. Of the three potential sources of conductive water at Wakulla Spring, seawater intrusion is the most probable source, based on the best available evidence at hand.

## Conclusion

This study provides strong evidence that seawater intrusion in a coastal karst aquifer is migrating 14 miles inland to one of Florida’s major first magnitude springs. The conceptual model established by previous researchers provides the mechanism by which seawater can enter the conduit system at SCSC and migrate northward against the prevailing regional potentiometric gradient to Wakulla Spring. The analysis of the conductivity data establishes that the increases in conductivity move from south to north over time, and the analysis of the geochemical data allows us to rule out both upwelling connate water and anthropogenic sources as the cause of the increased conductivity values observed by the 2D-ACM flowmeters. This is not only the first documented case of seawater intrusion at SCSC migrating to Wakulla Spring, but also the longest documented extent of seawater intrusion through subsurface conduit networks in the WKP, a typical coastal karst aquifer with conduit networks. While the increases in conductivity noted at the vent of Wakulla Spring are small in an absolute sense, they show that seawater intrusion and transport in karst aquifers are complex processes that can occur over extremely long distances.

More work is needed to provide further insights into the behavior of the local aquifer system southwest of Wakulla Spring. New karst window stations and monitoring devices would need to be installed in order to establish the east-west extent of seawater intrusion; at present it is unclear whether SCSC is a single point of intrusion or whether the data described in this paper are indicative of wider seawater intrusion. Continuous chemical observation of water sample at Wakulla Spring would also be helpful to confirm the results in this study and definitively link the conductivity increases at the Wakulla vent to coastal seawater. Advanced groundwater modeling approaches, such as density-dependent conduit-matrix hybrid numerical model, are also able to provide a quantitative estimation of seawater intrusion through the conduit networks. At present, however, the best available evidence at hand all point to the source of the rising conductivity values at the Wakulla vent being long distance seawater intrusion from the coastal SCSC system.

## Additional Information

**How to cite this article**: Xu, Z. *et al*. Long distance seawater intrusion through a karst conduit network in the Woodville Karst Plain, Florida. *Sci. Rep.* **6**, 32235; doi: 10.1038/srep32235 (2016).

## Figures and Tables

**Table 1:** The five highlighted periods of increased conductivity in the Wakulla conduits.

Period	Start	End	Length (days)
1	2007-05-15	2008-02-28	289
2	2008-04-15	2008-08-31	138
3	2011-05-13	2012-03-24	316
4	2012-05-02	2012-07-06	65
5	2012-10-31	2013-03-06	136

**Table 2:** Dates of rising conductivity in the Wakulla and SCSC conduits.

Station	Period 1	Period 2	Period 3	Period 4	Period 5
C	2007-08-18	2008-07-27	2011-09-05	No Data	2012-12-09
B	No Change	No Change	No Change	No Change	No Change
D	No Data	No Change	2012-01-12	No Change	2013-02-06
AD	2007-08-10	2008-07-01	2011-08-31	2012-06-22	2012-12-05
K	2007-08-10	2008-07-01	2011-08-31	2012-06-22	2012-12-05
AK	2007-08-10	2008-07-01	2011-08-31	2012-06-22	2012-12-05
Revell	No Data	No Data	2011-08-20	2012-05-25	2012-11-28
SCSC #1	No Data	No Data	2011-05-13	No Data	No Data
SCSC #10	No Data	No Data	2011-05-13	2012-05-02	2012-10-13
SCSC (USGS)	No Data	2008-04-15	No Data	No Data	No Data

Stations are arranged from north to south reading down the table. The SCSC (USGS) dataset is maintained and measured by USGS and NWFWMD at the mouth of SCSC embayment.

**Table 3:** Dates of return to nominal conductivity in the Wakulla and SCSC conduits.

Stations	Period 1	Period 2	Period 3	Period 4	Period 5
C	2008-02-15	2008-09-01	No Data	No Data	No Data
B	No Change	No Change	No Change	No Change	No Data
D	No Change	No Change	2012-02-22	No Change	2013-02-17
AD	2008-03-05	2008-09-01	2012-03-20	2012-07-05	2013-03-05
K	2008-03-05	2008-09-01	2012-03-20	2012-07-05	2013-03-05
AK	2008-03-06	2008-09-01	2012-03-20	2012-07-05	2013-03-05
Revell	No Data	No Data	2012-03-22	No Data	2013-03-16
SCSC #1	No Data	No Data	No Data	No Data	No Data
SCSC #10	No Data	No Data	2012-03-10	2012-06-29	2013-03-06
SCSC (USGS)	2008-02-23	2008-08-27	No Data	No Data	No Data

Stations are arranged from north to south reading down the table. The SCSC (USGS) dataset is maintained and measured by USGS and NWFWMD at the mouth of SCSC embayment.
